# Nanomicelle‐Assisted Targeted Ocular Delivery with Enhanced Antiinflammatory Efficacy In Vivo

**DOI:** 10.1002/advs.201700455

**Published:** 2017-11-10

**Authors:** Yu‐Hua Weng, Xiao‐Wei Ma, Jing Che, Chan Li, Juan Liu, Shi‐Zhu Chen, Yu‐Qin Wang, Ya‐Ling Gan, Hao Chen, Zhong‐Bo Hu, Kai‐Hui Nan, Xing‐Jie Liang

**Affiliations:** ^1^ Chinese Academy of Sciences (CAS) Center for Excellence in Nanoscience National Center for Nanoscience and Technology Beijing 100190 P. R. China; ^2^ Laboratory of Controllable Nanopharmaceuticals CAS Key Laboratory for Biomedical Effects of Nanomaterials and Nanosafety National Center for Nanoscience and Technology Beijing 100190 P. R. China; ^3^ College of Materials Science and Opto‐Electronic Technology University of Chinese Academy of Sciences Beijing 100049 P. R. China; ^4^ School of Ophthalmology and Optometry and Eye Hospital Wenzhou Medical University Wenzhou P. R. China

**Keywords:** antiinflammation, flurbiprofen nanomicelles, functional peptide, ocular delivery system

## Abstract

Ocular inflammations are common diseases that may lead to serious vision‐threatening obstacles. Eye drops for antiinflammation therapy need to be administered multiple times daily at a high dosage due to the rapid precorneal removal and low bioavailability of drugs. To overcome these problems, a cRGD‐functionalized DSPE‐PEG_2000_ nanomicelle (DSPE‐PEG_2000_‐cRGD) encapsulated with flurbiprofen is proposed. The tailored nanomicelles trigger specific binding to integrin receptors on the ocular surface, which leads to rapid and robust mucoadhesion, superior ocular surface retention, and transcorneal penetration behaviors of nanomicelles. Due to the enhanced drug delivery on ocular surface and in aqueous humor, the functionalized nanoformulation significantly improves ocular antiinflammation efficacy at a low dosage by blocking the synthesis of inflammatory mediators and cytokines. The present study demonstrates a promising strategy that uses a functional peptide combined with nanomicelles for targeted delivery to the eye in ophthalmologic applications.

## 1 Introduction

Ocular inflammation commonly occurs after eye injuries, infection, ocular surgery, and eye irritation and may lead to vision loss or blindness. Among various drug administration routes, topical ophthalmic solution is the most accessible and noninvasive. However, due to multiple clearance mechanisms such as solution drainage, turnover of tears and aqueous humor, and systemic absorption through conjunctiva, the efficiency of the drugs administered from a common ophthalmic solution is typically less than 5% and can be below 1%, suggesting the poor drug bioavailability.^1^, ^2^, ^3^, ^4^ As a result, many eye drops must be administered multiple times daily for long period. For example, the nonsteroidal antiinflammatory drug flurbiprofen was used for treatment of keratoconjunctivitis, postoperative ocular inflammation, prevention of cystoid macular edema, and ocular pain.^5^, ^6^, ^7^ Commercial flurbiprofen eyedrop (0.03% flurbiprofen sodium (FBNa), Ocufen, Allergan) is suggested to be administered 3–4 times daily for 2–3 weeks. The use of high‐dosage topical eye drops frequently brings toxic side effects and ocular surface impairments such as temporary blurred vision, inflammation of the ocular surface, dry eye, meibomian gland dysfunction, and chronic allergy.^8^, ^9^ Poor patient compliance often occurs when patients are treated with two or more topical ophthalmic solutions simultaneously.

To address these challenges, the application of nanotechnology is expected to lead to new perspectives in ocular disease management. Nanotechnology‐based ocular delivery systems can overcome many drawbacks of traditional ophthalmic drugs such as insolubility in water, eye irritation, and low drug administration efficiency.^10^ Nanomedicine with tailored properties can prolong the contact time of drugs on the ocular surface due to enhanced mucoadhesion and controlled drug release via precise carrier design.^11^, ^12^, ^13^ Many ocular delivery systems such as liposomes, micelles, dendrimers, in‐situ hydrogels, and nanoparticle‐containing contact lens have emerged.^11^, ^14^, ^15^, ^16^ Among these different delivery systems, nanomicelles have attracted attention in ocular applications because of their noticeable advantages such as easy fabrication, high drug‐loading potency, and small size for transcorneal penetration.^17^, ^18^, ^19^ An ideal nanomicelle‐based ocular delivery system combines the advantages of small size and a mucoadhesive surface with the ligand to realize targeted therapy.

Recently, the amphiphilic material DSPE‐PEG_2000_ has been explored with the aim of prolonging the residence time at the site of administration and improving therapeutic efficacy. For example, liposomes prepared with DSPE‐PEG_2000_ and helper lipids were a popular kind of nonviral ocular gene carrier.^20^, ^21^ DSPE‐PEG_2000_ contained liposomes with conjugated cRGD ligand were proved to be efficient vectors for retinal cells when loaded with VEGF‐siRNA.^22^, ^23^ DSPE‐PEG_2000_‐cRGD has also realized a combination therapy of two drugs in choroidal neovascularization).^24^ However, systematic evaluation of DSPE‐PEG_2000_‐cRGD as nanocarriers for anterior ocular disease therapy has not been reported before.

In this work, we designed a functionalized flurbiprofen (FBP)‐encapsulated DSPE‐PEG_2000_‐cRGD nanomicelles that showed rapid mucoadhesion on the ocular surface through the interaction of a cyclic peptide ligand c(GRGDSPKC) (cRGD) and the corneal epithelium, with long ocular surface retention time, and robust transcorneal penetration abilities. The nanomicelles significantly inhibited ocular inflammation progression whereas the commercial formulation FBNa failed at the same dosage, suggesting a reduced dosage of drugs may maintain effective therapeutics. The cornea targeting and ocular retention properties of the DSPE‐PEG_2000_‐cRGD nanomicelles were investigated systematically in vitro and in vivo. The nano delivery system proposed in our research solved the vital problems of targeted delivery, transcorneal penetration, and antiinflammation therapy in ocular disease management and has important implications for developing nanoformulations for ophthalmologic applications.

## 2 Results and Discussion

### 2.1 Preparation and Characterization of Nanomicelles

**Figure 1:**
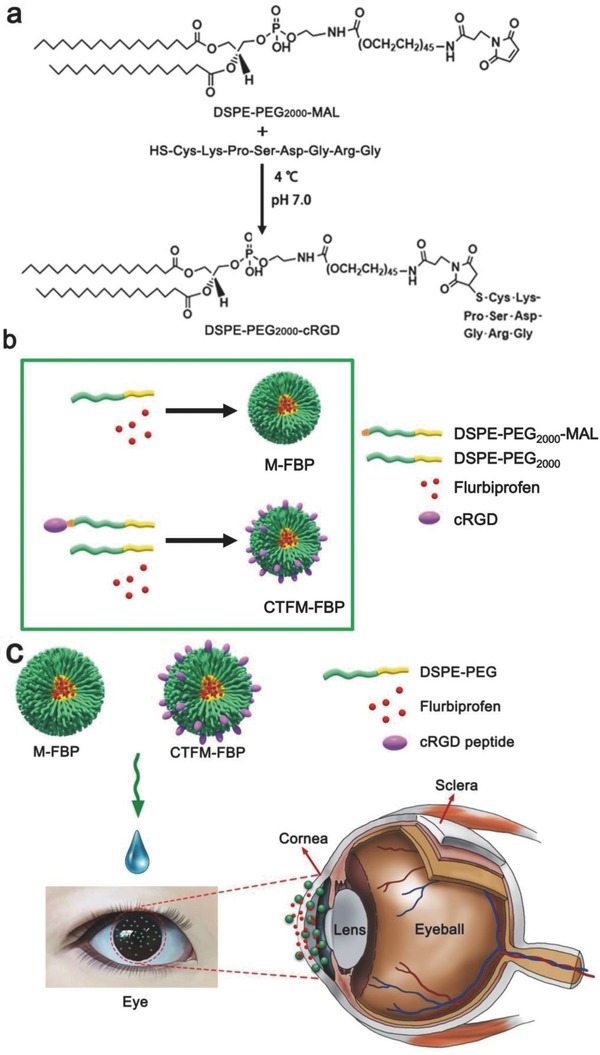
Nanocarrier synthesis and preparation of FBP‐loaded nanomicelles. a) DSPE‐PEG_2000_‐cRGD was synthesized by coupling the thiol group of the cRGD peptide with the maleimide group of DSPE‐ PEG_2000_‐MAL. b) Scheme of the preparation of M‐FBP and CTFM‐FBP nanomicelles. c) Schematic illustration of nanomicelle‐assisted targeted ocular delivery.

**Figure 2:**
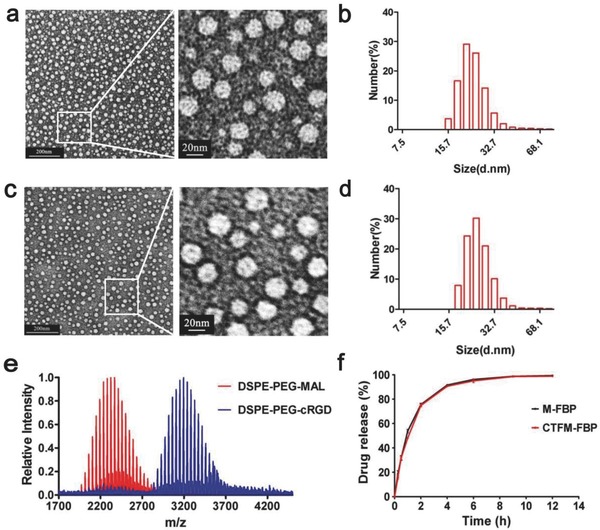
Characterizations of nanomicelles. a) Transmission electron microscopy (TEM) images of M‐FBP. b) Dynamic laser scanning (DLS) measurement of M‐FBP. c) TEM images of CTFM‐FBP. d) DLS measurement of CTFM‐FBP. e) MALDI–TOF–MS analysis of conjugation of the cRGD peptide with DSPE‐PEG_2000_‐MAL. f) Time course of FBP release from M‐FBP and CTFM‐FBP nanomicelles in artificial tears over 12 h.

The amphiphilic polymer 1,2‐distearoyl‐*sn*‐glycero‐3‐phosphoethanolamine‐*N*‐[methoxy (polyethylene glycol)‐2000] (DSPE‐PEG_2000_) was a widely used pharmaceutical and medical material which could form micelles easily.^25^ In this work, DSPE‐PEG_2000_ was used to prepare flurbiprofen (FBP)‐loaded nanomicelles (M‐FBP) and cornea‐targeting peptide‐functionalized nanomicelles (CTFM‐FBP). The synthesis of DSPE‐PEG_2000_‐cRGD and preparation of nanomicelles are outlined (**Figure** **1**). With the classic thiol–maleimide coupled reaction under pH 7.0, the cRGD peptide was successfully coupled with 1, 2‐distearoyl‐*sn*‐glycero‐3‐phosphoethano‐ lamine‐*N*‐[maleimide (polyethylene glycol)‐2000] (DSPE‐PEG_2000_‐MAL) and the matrix‐assisted laser desorption/ionization time‐of‐flight mass spectrometry (MALDI–TOF–MS) analysis showed that the mass peak was right‐shifted 800 Da after the coupling process (**Figure** **2**e). The nanomicelles were formed using the solvent evaporation method.^26^ As the transmission electron microscopy and dynamic light scattering (DLS) revealed, both the M‐FBP and CTFM‐FBP nanomicelles had homogenous spherical shapes that were ≈19 nm (19.1 ± 1.8 nm and 19.3 ± 2.1 nm, respectively; Figure 2a,d). The ζ potentials of the M‐FBP and CTFM‐FBP were −25.2 ± 1.0 and −22.0 ± 0.7 mV, respectively. We also investigated the drug‐loading content and drug encapsulation capacity of both formulations. Both the M‐FBP and CTFM‐FBP had high drug‐loading efficiencies and encapsulation capacities (Table S1, Supporting Information).

The stabilities of M‐FBP and CTFM‐FBP nanomicelles in water solution and artificial tears were studied, respectively. When nanomicelles were stored in water solution for 3 months under 4 °C, no significant changes were found in particle size and ζ potential after storage. Only when stored for 3 month under 25 °C, particle size gradually increased to above 40 nm. However, when nanomicelles were stored in artificial tears for 1 week at 25 °C, the average size of nanomicelles increased up to 60 nm and particle aggregation happened. No significant changes were found in particle size after storage under 4 °C for 1 week (Figure S1, Supporting Information). The drug encapsulation capacities of nanomicelles were not affected regardless of storage in water or artificial tears in both 4 and 25 °C. As we can find, the stabilities of M‐FBP and CTFM‐FBP nanomicelles are the same. It seems that the nanomicelles are less stable in artificial tears than water solution, probably because of the content of salts (phosphate buffer saline and CaCl_2_), bovine serum albumin (BSA), and lysozyme in artificial tears affected the size of the micelles.

### 2.2 Drug Release in Artificial Tears

For topical delivery to the anterior segment, due to the specific anatomy of the eye and fast clearance mechanisms, the balance between the drug release from nanocarriers and rapid precorneal clearance mechanisms is vital. If the drug is released too rapidly, toxicities may appear and the drugs may be cleared quickly; however, slow drug release may compromise the therapeutic efficacy because of ineffective drug concentration. Therefore, we investigated the FBP release profile of M‐FBP and CTFM‐FBP in artificial tears. As shown in Figure 2f, in the first half‐hour, 30% FBP was released and 50% FBP was released at 1 h. Most of the drugs were released (more than 90%) from the nanocarriers within 4 h. Because of their similar structures, M‐FBP and CTFM‐FBP released FBP in a similar way.

### 2.3 Specific Mucoadhesive Interactions of Functionalized Nanomicelles with Corneal Epithelial Cells

**Figure 3:**
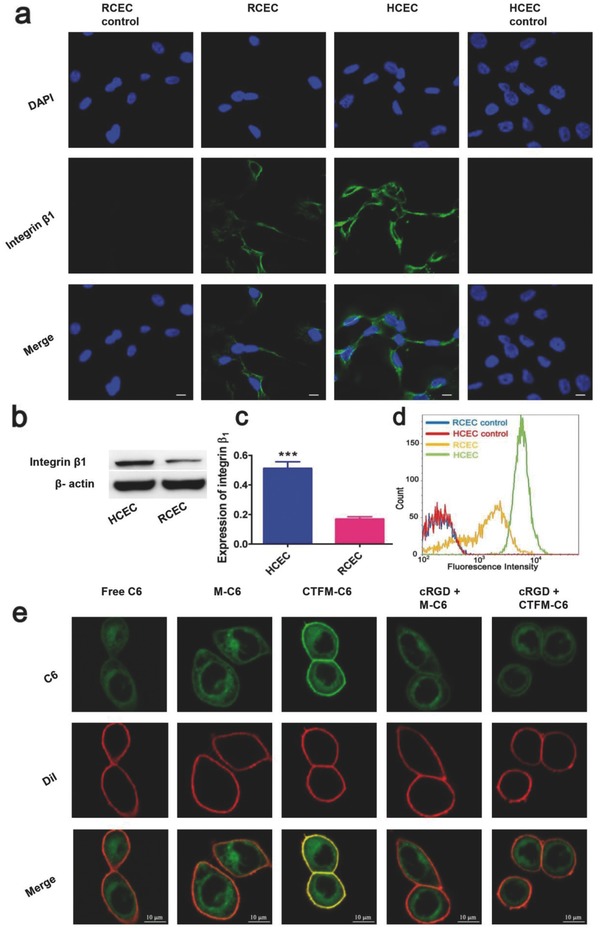
Identification of integrin β1 expression in human corneal epithelial cells (HCECs) and primary rabbit corneal epithelial cells (RCECs). a) Laser confocal microscopy images (scale bar: 10 µm). b) Representative and c) Quantitative western blot analysis of integrin β1 expression, in which the lanes represent the integrin β1 or β‐actin level of HCECs and RCECs. Data are presented as mean ± s.e.m. (*n* = 5). ***P < 0.001, unpaired Student's *t*‐test. d) Flow cytometry analysis of integrin β1 expression using FITC‐labeled antiintegrin β1 antibody with HCECs and RCECs. e) In vitro nanomicelle binding assay after culturing nanomicelles with HCECs for 2 min. The coumarin 6 (C6) was encapsulated into nanomicelles instead of FBP to provide green fluorescence. Cell membranes were stained with Dil fluorescence probe (Red) (scale bar: 10 µm).

The colloidal nanosystems are attractive ocular delivery carriers because of their mucoadhesive interaction with the mucosal surface of the eye. However, this interaction is not sufficient to allow for the transcorneal penetration of particles and lacks specificity. The ocular surface is covered by two highly specialized tissues: the conjunctival epithelium and the corneal epithelium. The corneal epithelium, the most important part of the corneal barrier, is composed of multilayers of corneal epithelial cells. To study the mucoadhesive interaction of M‐FBP and CTFM‐FBP with the corneal surface, we selected human epithelial cells (HCECs) for the following research. First, we confirmed that integrin β1 was overexpressed in HCECs and primary rabbit corneal epithelial cells (RCECs) and that HCECs showed much higher expression (**Figure** **3**). From laser scanning confocal microscopy images, we noticed that integrin β1 receptors were primarily distributed on cell membranes and at cell junctions. Quantitative analyses such as flow cytometry and western blot also validated the high expression of integrin β1 in HCECs and RCECs.

We then investigated the specific mucoadhesive behaviors of cRGD‐functionalized nanomicelles using corneal epithelial cells in an in vitro nanomicelle binding assay. To facilitate observation, FBP was replaced with coumarin‐6 (C6), which gives green fluorescence. Considering the rapid clearance mechanisms of most eye drop formulations, we cocultured HCECs with different C6 formulations for 2 min. Cells were also precultured with several endocytosis inhibitors to reduce the cellular uptake of C6 as much as possible. As shown in Figure 3e, after a short culture time, the functional CTFM‐C6 nanomicelles showed stronger cell adhesive features than free C6 and M‐C6, which showed minimal adherence on cell membranes. When the cells were pretreated with excess cRGD peptide, the adhesive behavior of CTFM‐C6 nanomicelles on the cell membrane was greatly inhibited. The results revealed that CTFM‐C6 could target corneal epithelial cells quickly via integrin–cRGD interactions.

### 2.4 In Vivo Ocular Surface Retention of Functionalized Nanomicelles

**Figure 4:**
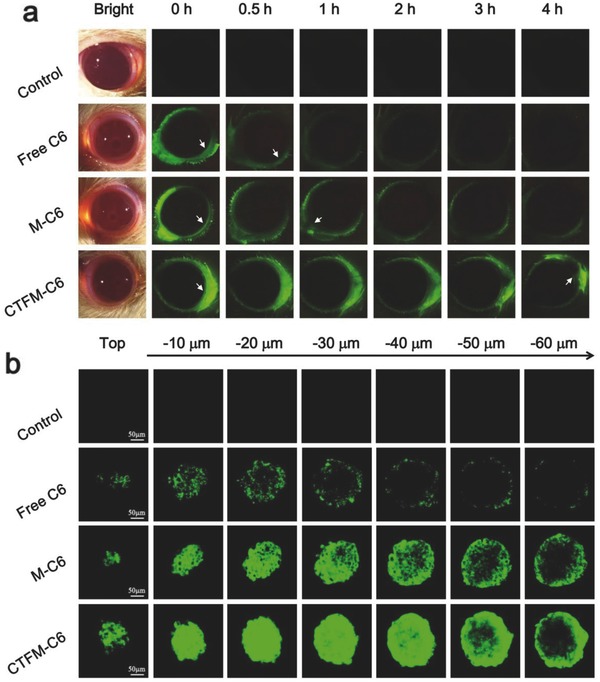
Ocular surface retention and transcorneal penetration studies. a) Fluorescence microscopy of rats' eyes after treated with different C6 formulations. The white arrows show typical drug retention sites. b) Transcorneal penetration study in 3D cultured multilayer HCEC spheroids. After incubating the spheroids in different C6 formulations for 4 h, confocal microscopy images were taken by scanning the spheroids from top to bottom with 10 µm depth per image. The scale bar is 50 µm.

Fluorescence imaging was used to evaluate the retention of different C6 formulations on ocular surface (**Figure** **4**a; Figure S2, Supporting Information). From the fluorescence images we could find that free C6 was rapidly cleared out by ocular surface within 0.5 h. The retention time of unmodified nanomicelles (M‐C6) on ocular surface was prolonged to 0.5–1 h while fluorescence could still be observed after 1 h. The cRGD modified nanomicelles (CTFM‐C6) showed prolonged drug retention time for as long as 4 h after the initial instillation. It is noteworthy that the average fluorescence intensities of CTFM‐C6 at 4 h was still more than the average fluorescence intensities M‐C6 at 1 h. Significant differences were found between M‐C6 and CTFM‐C6 at all time points. Considering the rapid clearance rate of normal eye drops within a few minutes, our results proved that the cRGD modified functionalized nanomicelles can significantly prolong the drug retention time on ocular surface.

### 2.5 TransCorneal Penetration Efficiency of Functionalized Nanomicelles through 3D Multilayer Corneal Epithelial Cell Spheroids

A main reason why ophthalmic solutions exhibit low drug bioavailability is prevention of molecule penetration by the corneal epithelium. The corneal epithelium is ≈50 µm thick, which could significantly limit transcorneal penetration of particles as small as 21 ± 1 nm.^27^, ^28^ To observe the penetration process of nanomicelles across the corneal epithelium, we constructed a 3D multilayer corneal epithelial cell spheroid ≈200 µm in diameter to test the penetration ability of CTFM‐C6. Confocal microscopy was used to confirm the exact location of C6 in the spheroids by scanning them from top to bottom with a 60 µm depth. As shown in Figure 4b, free C6 was only observed on the spheroid surface with a sporadic dotted distribution. M‐C6 nanomicelles were distributed in the outer region and in the first few layers of the spheroid. The CTFM‐C6 penetrated the deep inner region of the spheroid with a much more homogeneous distribution. Quantitative analysis for penetration distances of different C6 formulations revealed that CTFM‐C6 penetrated deeper into the inner region of the HCEC spheroid and showed 3.7 and 17 times more than M‐C6 and free C6, respectively (Figure S3, Supporting Information). These data clearly demonstrated that CTFM‐C6 exhibited much better transcorneal penetration than nonspecific M‐C6 and free C6 due to the targeted ligand and small size.

### 2.6 Ocular Antiinflammation Efficiency of Functionalized Nanomicelles

**Figure 5:**
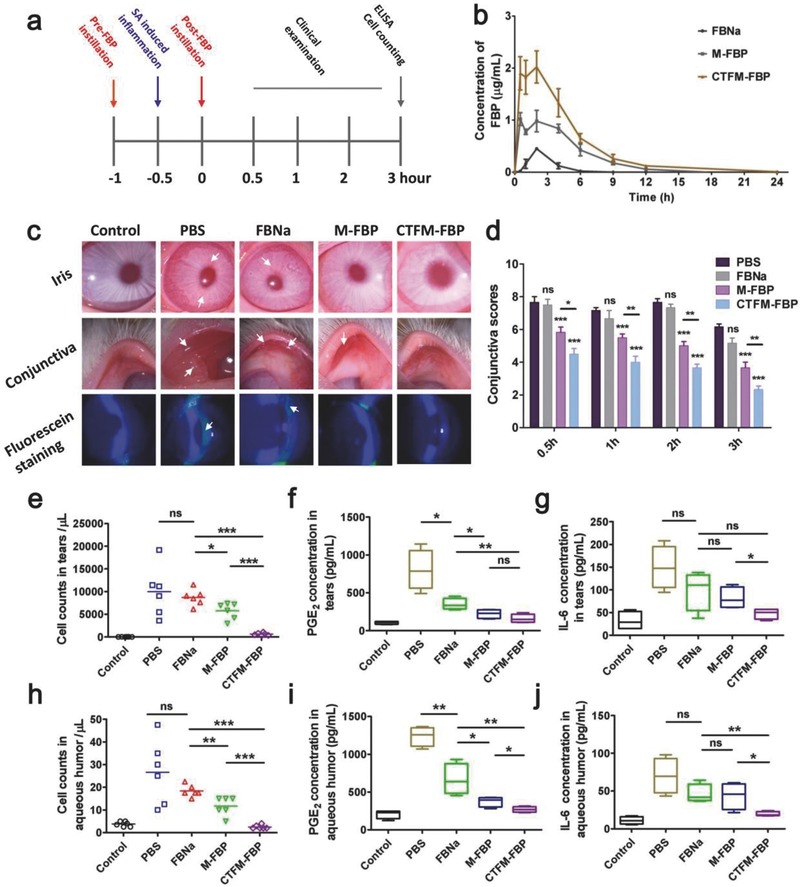
In vivo antiinflammation efficiency of FBP formulations and single‐dose pharmacokinetics study. a) Timeline of drug administration and the antiinflammation study. b) Intraocular pharmacokinetic profile of FBP in aqueous humor after one dose of an FBP formulation. Data are mean ± s.e.m. (*n* = 4). c) Clinical symptoms of ocular inflammation such as conjunctiva congestion, swelling, and iris hyperemia were examined using a slit lamp. Fluorescein staining was used for examination of corneal epithelial integrity. White arrowheads indicate the inflammatory sites over the conjunctiva, iris, and cornea. d) Clinical scores of ocular inflammation in the conjunctiva of rabbit eyes. Statistical analysis of two‐way ANOVA with repeated 3 measures was conducted by comparing with the PBS group (*n* = 6). *P < 0.05, **P < 0.01, ***P < 0.001. e) Polymorphonuclear leukocytes (PMN) in tear fluid and h) PMN in aqueous humor, quantified using a hemocytometer. f,i)The prostaglandin E_2_ (PGE_2_) and g,j) cytokine IL‐6 in tear fluid and aqueous humor were measured using ELISA. Student's *t*‐tests were conducted in panels (e)–(j). The data are presented as the mean ± s.e.m (*n* = 6). *P < 0.05, **P < 0.01, ***P < 0.001. “ns” represents no statistical difference.

To investigate the antiinflammation efficiency of FBP nanomicelles, we built a noninfectious ocular inflammation model using New Zealand albino rabbits. 1% sodium arachidonate (w/v) was used to induce ocular inflammation and inflammatory symptoms were quantified 0.5–3 h after the last instillation of FBP formulations (**Figure** **5**a). The negative control groups for PBS showed no antiinflammation effect, which resulted in severe conjunctiva redness and swelling, iris hyperemia, and high clinical scores during the quantification period (Figure 5d; Table S2, Supporting Information). Many polymorphonuclear (PMN) leukocytes accumulated in the tear fluid and in aqueous humor at 3 h. The commercially used FBNa formulation resulted in limited inhibition of ocular inflammation symptoms, the PMN leukocytes amounts were reduced in the tear fluid and in aqueous humor. However, slightly swollen conjunctiva and iris hyperemia still occurred, as well as damaged corneal epitheliums. When the inflamed eyes were treated with nanomicelle formulations, intact corneal epitheliums were found in the M‐FBP and CTFM‐FBP groups, suggesting dramatically reduced inflammation symptoms. However, there were still slight conjunctiva swelling symptoms in the M‐FBP group. Pleasingly, CTFM‐FBP exhibited better therapeutic efficiency than M‐FBP and showed minimum clinical scores and PMN leukocytes in the tear fluid and aqueous humor.

Prostaglandin E_2_ (PGE_2_), a derivative of arachidonic acid, is a mediator in ocular inflammation that can induce blood–aqueous barrier breakdown.^29^, ^30^ Increased PGE_2_ is produced in several pathologic conditions including inflammation and tissue injury. Here, the production of PGE_2_ in ocular inflammation was measured using ELISA. After the induction of ocular inflammation, PGE_2_ significantly increased in tear fluid and aqueous humor. However, its concentration was greatly decreased both in tears and in aqueous humor after treatment with CTFM‐FBP, which was significantly lower than those of the FBNa‐ and M‐FBP‐ treatment groups. These results verified that CTFM‐FBP could efficiently block the synthesis of PGE_2_ during the development of ocular inflammation. In addition to PGE_2_, inflammation mediators can induce the release of other inflammatory cytokines such as IL‐6. Several previous studies demonstrated that IL‐6 plays an important role in ocular inflammation.^31^, ^32^, ^33^, ^34^ In this study, we found that production of IL‐6 increased 4.6 and 6.3 times in tears and aqueous humor, respectively, after ocular inflammation was induced. When treated with CTFM‐FBP, its concentration was significantly reduced compared with treatment with FBNa or M‐FBP, suggesting that CTFM‐FBP reduced the release of ocular‐inflammation‐related cytokines more effectively than M‐FBP and FBNa. Considering the high dosage of clinical FBNa eye drops, CTFM‐FBP could inhibit ocular inflammation effectively at a significantly lower dosage.

To further understand the accumulation and transcorneal penetration behavior of CTFM‐FBP, we next investigated its in vivo pharmacokinetic profiles in aqueous humor after a single topical instillation. The area under the curve (AUC_0→24 h_) for FBNa, M‐FBP and CTFM‐FBP was 1.10 ± 0.28, 6.27 ± 1.24, and 11.50 ± 1.95 µg mL^−1^ h, respectively. One phenomenon needs to be paid attention was that M‐FBP also resulted in a significant elevation of *C*_max_ and AUC_0→24h_ in aqueous humor compared with FBNa formulation (Figure 5b; Table S3, Supporting Information). This is probably because of the mucoadhesive feature of DSPE‐PEG_2000_ and the small‐sized nanomicelles facilitated transcorneal penetration of delivered drugs. Meanwhile, the cRGD motif‐conjugated CTFM‐FBP exhibited a 1.83‐fold increase of AUC_0→24 h_ compared with M‐FBP (*P* < 0.01) and 10.45‐fold increase compared with the FBNa formulation (*P* < 0.001). The relative bioavailability (Fr%) of CTFM‐FBP was 10.45‐fold higher than the FBNa formulation. The enhanced AUC_0→24 h_ and *C*_max_ for CTFM‐FBP could be attributed to the targeted ligand on its surface and small size. With the mucoadhesive interaction between cRGD motif‐conjugated nanomicelles and the ocular surface, the retention time of FBP on cornea was further increased. The accumulated FBP on the corneal surface facilitated more FBP penetration across the cornea into inner side of the eye.

### 2.7 Ocular Tolerance and Irritancy Study

**Figure 6:**
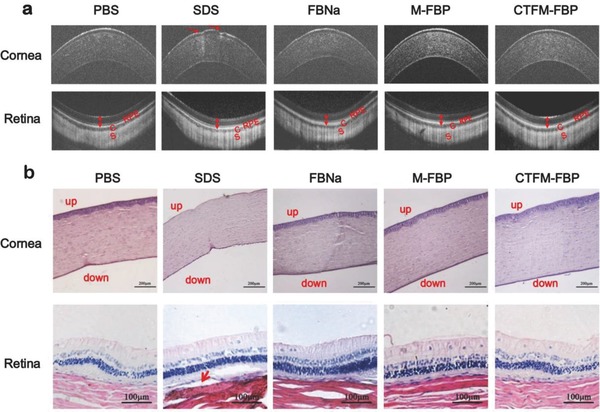
Histological examination of the cornea and retina of rabbit eyes after multiple instillations of FBP formulations. a) Representative optical coherence tomography (OCT) images of corneas and retinas after eyes were treated with the irritant 1% SDS, FBNa formulation, M‐FBP, or CTFM‐FBP 12 times. OCT imaging was conducted 24 h after the last instillation. Red arrows show corneal epithelium defects. Double sided arrows show the boundaries of the retina. “RPE” indicates the retinal pigment epithelium. The “C” represents the choroid and “S” represents the sclera. b) H&E staining of excised rabbit corneas and retinas at 24 h after the last instillation. Corneal cross sections show the epithelium, stroma, and endothelium (scale bar: 200 µm) and the red arrow shows retinal detachment (scale bar: 100 µm) (*n* = 3).

To assess the ocular safety of the FBP nanomicelles, we performed optical coherence tomography (OCT) and histological studies using hematoxylin–eosin (H&E) staining with New Zealand albino rabbits. A custom‐built ultrahigh resolution OCT (UHR‐OCT) instrument was applied to measure the central corneal and retina thickness after 12 instillations of different formulations. As we could see from **Figure** **6**, the irritant 0.5% sodium dodecyl sulfate (SDS) (w/v) resulted in severe lamellar corneal epithelium defects after the last instillation at 24 h. No significant difference of central corneal thickness was found in the CTFM‐FBP‐, M‐FBP‐, and FBNa‐treated groups. The intraretinal layer structures of the retina were clearly observed with layer boundaries in all groups. The retinal thickness of the CTFM‐FBP‐, M‐FBP‐, and FBNa‐treated groups was not affected.

In addition, histopathologic examination of corneal cross sections showed that, most of corneal epitheliums were detached from cornea when the eye was exposed to SDS (Figure 6). Some epithelial cells were also detached from the cornea in the FBNa‐treated group. In the M‐FBP‐ and CTFM‐FBP‐treated groups, intact and unaffected corneal structures were observed. This is probably due to the protection effect of DSPE‐PEG_2000_ carriers, as nanomicelle structures can protect the encapsulated payloads from rapid release and prevent exposure of the eye tissues to large amounts of drugs, therefore reducing dose‐dependent drug toxicity.^35^, ^36^ The H&E staining of retina showed that, except for slight retinal detachment in the SDS group, all the retinal architectures were well organized when eyes were treated with the other formulations. No apparent defects or inflammatory exudates were found in the outer nuclear layer, inner nuclear layer, or the retinal pigment epithelium (RPE) of the retina. These results are in accordance with the OCT imaging observations and suggest good intraocular tolerance of FBP nanomicelles.

The potential ocular irritancy was also examined using a slit lamp and scored according to a modified Draize test.^37^ After instillation of the irritant SDS, conjunctiva discharge, congestion, swelling, and iris hyperemia lasted for more than 6 h (Table S4, Supporting Information). Temporary irritation of the conjunctiva was found in two of three rabbits 10 min after instillation of the FBNa formulation, followed by a recovery at 6 h. Similar symptoms were found in one of three rabbits 10 min and 6 h after instillation of CTFM‐FBP and M‐FBP. No other clinically abnormal symptoms were observed after 24 h in all groups, which suggested that the slight irritation caused by CTFM‐FBP nanomicelles was temporary.

Topical ophthalmic solutions are the most common formulations for anterior ocular disease therapy. However, they are commonly administered multiple times daily due to rapid drug clearance and failure to target the cornea.^1^, ^2^, ^3^ Mucous‐targeting molecules such as chitosan have been studied because their positive charge could interact with the negatively charged mucin on the corneal surface through electrostatic interactions. However, the interactions may be greatly disturbed by counter‐ions in tears and by tear turnover.^38^, ^39^ To further strengthen the mucoadhesive properties of nanosystems, one strategy is the use of a covalent binding reaction between specific ligands and the corneal surface. For example, phenylboronic acid (PBA)‐modified nanoparticles could adhere to the ocular mucous membrane through covalent linkage between PBA and sialic acid.^40^ Another optimal strategy is locating a receptor that is distributed on the ocular surface and developing a corresponding ligand that could be linked with drugs. Using this strategy, a targeted polymeric micellar system that can deliver hydrophobic ophthalmic drugs such as flurbiprofen to the ocular surface and interact with widely expressed integrin was developed in our study.

Integrins are transmembrane receptor proteins that play essential roles in cell survival, migration, differentiation, and proliferation.^41^ They can recognize and bind to specific ligand domains. For example, integrin β_1_ (α_2_β_1_, α_3_β_1_, α_5_β_1_, and α_ν_β_1_), which are widely expressed in cornea, can bind to the three amino acid sequence arginine–glycine–aspartic acid (RGD).^42^, ^43^ According to literatures, the RGD–integrin system plays a central role in the corneal epithelial wound healing process.^44^, ^45^ The attachment of corneal epithelial cells to fibronectin was inhibited by the addition of excess synthetic GRGDS peptide in a concentration‐dependent manner, suggesting that integrins on corneal epithelial cells specifically bind to the RGD domain of fibronectin.^46^

During topical administration, transcorneal penetration is the dominant way for drug transportation to intraocular tissues. Two main factors are involved in nanoparticle transcorneal penetration: size and interactions with the ocular surface. It has been reported that the cornea is impermeable to molecules larger than 5 kDa.^47^ Previous study has shown that nanoparticles can interact with the ocular surface more effectively than linear polymers.^48^ Small nanoparticles (<100 nm) should distribute more uniformly throughout the ocular surface whereas microparticles tend to accumulate in the lower sac of the conjunctiva due to gravitational forces. Although small nanoparticles show advantages in drug delivery, their usage in ocular topical administration is still limited because of rapid clearance. The cRGD ligand introduced on the particle surface confers the integrin receptor‐mediated interaction with the ocular surface. The interaction promotes the mucoadhesive properties and prolongs the retention time of the nanosystem on ocular surface, therefore ensuring the transcorneal penetration behavior. Based on the in vitro and in vivo studies, we believe that the robust mucoadhesion and small size of CTFM‐FBP leads to improved treatment of ocular inflammation at a low dosage and with high bioavailability of FBP.

In this study, we investigated an original functionalized supramolecular nanomicelle system to deliver the ocular antiinflammation drug FBP by topical instillation. The resulting nanoformulation effectively prolonged drug retention on ocular surface, enhanced the transcorneal penetration efficiency and led to a better ocular antiinflammation effect. Several favorable features make our nanoformulation a promising ophthalmologic option, including (i) an amphiphilic property with both hydrophilic and hydrophobic character, which improves delivery of poorly water‐soluble ocular drugs; (ii) small size (19 nm) with a narrow size distribution. The small DSPE‐PEG nanomicelles ensure homogenous distribution on the corneal surface and are beneficial for deeper penetration into intraocular tissues; (iii) a rapid and robust mucoadhesive property mediated by the integrin–RGD interaction. The conjugation of the specific cRGD ligand to the nanomicelle surface promotes long time drug retention on the ocular surface and facilitates transcorneal penetration of drugs; (iv) balanceable drug release that leads to sustained and effective drug concentration on the ocular surface and in intraocular tissue without causing toxicity to normal tissue; (v) enhanced drug bioavailability by enriching drug accumulation on the corneal surface and reducing drug removal through clearance mechanisms, ensuring better antiinflammation therapy at a low dosage by blocking synthesis of inflammatory cytokines and mediators; and (vi) good ocular tolerance even when instilled many times. The sustained drug release from CTFM‐FBP nanomicelles avoids exposure of ocular tissues to large amounts of free drug compared with free FBP. Moreover, the DSPE‐PEG nanocarrier is an FDA‐approved biomedical material. Based on these advantages, this nanomicelle‐based CTFM‐FBP may have great potential in nanomedicine for clinical transformation in ocular disease therapy.

## Conflict of Interest

The authors declare no conflict of interest.

## Supporting information

Supplementary
