# Supplementary material for: Nanomicelle‐Assisted Targeted Ocular Delivery with Enhanced Antiinflammatory Efficacy In Vivo
Source: Adv Sci (Weinh). 2017 Nov 10;5(1):1700455. doi: 10.1002/advs.201700455 (PMC5770669; doi:10.1002/advs.201700455)
Supplement: Supplementary file 1 — Supplementary [file ADVS-5-na-s001.pdf]

Copyright WILEY-VCH Verlag GmbH & Co. KGaA, 69469 Weinheim, Germany, 2017.

ADVANCED SCIENCE
Open Access

Supporting Information

for *Adv. Sci.,* DOI: 10.1002/advs.201700455

Nanomicelle-Assisted Targeted Ocular Delivery with Enhanced Antiinflammatory Efficacy In Vivo

Yu-Hua Weng, Xiao-Wei Ma, Jing Che, Chan Li, Juan Liu, Shi-Zhu Chen, Yu-Qin Wang, Ya-Ling Gan, Hao Chen, Zhong-Bo Hu, Kai-Hui Nan,* and Xing-Jie Liang**

Supporting Information

Nanomicelle-assisted targeted ocular delivery with enhanced anti-inflammatory efficacy *in vivo*

Yu-Hua Weng[abcd+], Xiao-Wei Ma[abc+], Jing Che[abcd], Chan Li[abc], Juan Liu[abc], Shi- Zhu Chen[abc], Yu-Qin Wang[e], Ya-Ling Gan[abc], Hao Chen[e], Kai-Hui Nan[e]*, Zhong-Bo Hu[d]*, Xing-Jie Liang[abc]*

Materials and Methods

Materials

The flurbiprofen was obtained from J&K Scientific Ltd., China. 1,2-distearoyl-*sn*-glycero-3-phosphoethanolamine-N- [methoxy (polyethylene glycol)-2000] (DSPE-PEG_{2000}) and 1,2-distearoyl-*sn*-glycero-3-phosphoethanolamine-N-[maleimide(polyethylene glycol)-2000](DSPE-PEG_{2000}-MAL) were obtained from Nanocs Laysan Co. Ltd., USA. Chlorpromazine, cytochalasin D, dynasore hydrate, genistein, sodium arachidonate, lysozyme and bovine serum albumin were purchased from Sigma-Aldrich. Coumarin 6 was obtained from Aladdin Scientific Ltd. (Shanghai, China). Flurbiprofen sodium was purchased from Shenyang Coupling Biomedical Technology Co., Ltd. (Shenyang, China). DAPI and DiI were obtained from Molecular Probes Inc. (Eugene, USA). The cyclic Octapeptide c(Gly-Arg-Gly-Asp-Ser-Pro-Lys-Cys) (cGRGDSPKC) was obtained from Shanghai Top-peptide Biotechnology Co., Ltd. (China). All other regents were analytic grade and were used as received.

Preparation of flurbiprofen-loaded nanomicelles

The DSPE-PEG-cRGD was synthesized first. Briefly, peptide and DSPE-PEG_{2000}-MAL (wt/wt, 1:5) were dissolved in 50 mM HEPES buffer (pH 7.0). After stirring at 4 ℃ for 48 hours, the thiol group (-SH) of cysteine could selectively react with the maleimide group of DSPE-PEG_{2000}-MAL. The final product was dialyzed against deionized water for 24 hours with a 2 KDa molecular weight cut-off dialysis bag. The identification of DSPE-PEG-cRGD was performed using matrix-assisted laser desorption/ionization time-of-flight mass spectrometry (MALDI-TOF-MS, Bruker Daltonics, USA).

To prepare the flurbiprofen-loaded nanomicelles (M-FBP), the DSPE- PEG_{2000} and FBP (10:1, wt/wt) mixture was dissolved in 0.2 mL tetrahydrofuran. The mixture was added into 2 mL water and stirred overnight. The tetrahydrofuran was removed completely by vacuum rotary evaporation.

Free drugs were excluded by dialyzing the micelle solution against water through dialysis membrane tubes (molecular weight cut-off 6-8 KDa, Millipore) (Millipore, USA). The preparation process for the cornea-targeting peptide-functionalized nanomicelles (CTFM-FBP) was the same as that for M-FBP except that the DSPE- PEG_{2000} was replaced with a mixture of DSPE- PEG_{2000} and DSPE-PEG-cRGD (4:1, wt/wt). For the *in vitro* confocal microscopy and *in vivo* fluorescence microscopy, the fluorescence probe coumarin 6 was used as a model drug and was loaded into micelles using the above procedure.

Characterization of the nanomicelles

The amount of FBP in the nanomicelles was quantified using a high-performance liquid chromatograph (HPLC) system (CBM-20A, Shimazu, Japan) equipped with a UV detector. A C_{18} column with a guard column was utilized. The mobile phase was acetonitrile and KH_2PO_4 (pH 4.5, 0.01 M) buffer (55:45, vol/vol) at a flow rate of 1 mL/min and a 250 nm detection wavelength. The drug-loading capacity and drug encapsulation efficiency were calculated using the following formula:

$$\text{drug-loading capacity (DLC, \%)} = W_1/W_2 \times 100\ \%$$

$$\text{drug encapsulation efficiency (EE, \%)} = W_1/W_0 \times 100\ \%$$

where W_1 is the amount of FBP that was loaded into nanomicelles, W_2 is the total amount of drug loaded nanomicelles, and W_0 is the initial fed amount of FBP. Morphology and size characterizations of the nanomicelles were conducted using low-acceleration-voltage TEM (HT7700, Hitachi). 5 μL of each micelle solution (micelle concentration of 0.5 mg/mL) was dropped onto a carbon film-supported copper grid. After the samples were half-air dried, 1 % (wt/vol) uranyl acetate was used to stain the samples for 5-10 minutes. The excess staining reagent was removed and followed air-drying before TEM observation. The zeta potential and size distribution of the nanomicelles were determined using dynamic light scattering (DLS, Nano-ZS, Malvern).

Stability studies

The physical stability of M-FBP and CTFM-FBP nanomicelle solution (water solution) was evaluated after storage for 3 months under different temperature. Exact volumes of each solution was stored in glass bottles and placed at 4 and 25 ℃. After stored for 1 and 3 months, the particle size, morphology, zeta potential and drug encapsulation efficiency were determined.

For the stability study of nanomicelles in artificial tears, both M-FBP and CTFM-FBP

nanomicelles were firstly dispersed in artificial tears (FBP concentration: 0.3 mg/mL). The size, morphology, zeta potential and drug encapsulation properties of the nanomicelles were analyzed after different storage time (1, 4, 7 days) at 4 and 25 □.

***In vitro* drug release in artificial tears**

The artificial tears were prepared with PBS buffer (0.01 M), 0.03 mM $CaCl_2$, 1 mg/mL lysozyme and 0.1 mg/mL bovine serum albumin.[1] The release of FBP from nanomicelles was performed with dialysis membrane tubes (molecular weight cut-off 6-8 KDa, Millipore). First, the nanomicelle solution was dispersed in artificial tears. Then, 150 μL nanomicelle solution was transferred to each dialysis membrane tube. The tubes were put in a container filled with 2 L artificial tears and stirred at 37 □ for 12 hours. At 0.25, 0.5, 1, 2, 4, 6, 9 and 12 hour time points, three tubes were collected and the residual FBP in each tube was measured using the HPLC method mentioned above. The released FBP was calculated using the initial fed FBP minus the residual FBP in each tube.

Cell culture

The human corneal epithelial cell line (HCEC) HCE-2 was purchased from the American Type Culture Collection and was tested negative for mycoplasma before use (Mycoplasma Detection Kit, Lonza). The primary rabbit corneal epithelial cells (RCECs) were obtained according to a modified Chan and Haschke's method.[2] Briefly, eyes from New Zealand albino rabbits (1.5 Kg) were enucleated and placed in sterile PBS buffer with 400 units/mL penicillin and 400 μg/mL streptomycin for 30 min. Then, the corneal epithelium was peeled from the stroma and along the limbus with an iris restorer and fine forceps. The dissected corneal epithelium was cut into small pieces and incubated with 0.05 % trypsin (Gibco, USA) for 30 min at 37 □. The cells were harvested and washed by centrifugation at 1000 rpm for 5 min. All steps were performed under sterile conditions. The culture media for the HCEC and RCEC cells were the same, including a 1:1 mixture of Dulbecco's modified eagle medium (DMEM) and Ham's F12 medium (Gibco), 5 μg/ml insulin (Sigma), 100 ng/mL hydrocortisone (J&K Co.), 5 ng/mL epidermal growth factor (Invitrogen, CA), 100 unit/mL penicillin, 100 μg/mL streptomycin, and 10 % fetal bovine serum (Gibco). All cells were cultured in an incubator at 37 □ with 5 % CO_2.

Immunofluorescence

The immunofluorescence methodology was used to determine the expression of integrin β_1 on

the HCECs and RCECs (n = 3). First, HCECs and passage 3 RCECs were cultured in complete medium to 70 % confluence. Then, the cells were fixed with 4 % paraformaldehyde for 10 min, washed with PBS buffer 3 times, and blocked with 10 % BSA for 1 hour at room temperature to block nonspecific conjugation sites. The cells were incubated with primary monoclonal mouse anti-integrinβ_1 antibody (1:50, ab30394, Abcam, USA) at 4 ℃ overnight. After washing with PBS 3 times, the cells were incubated with secondary FITC-conjugated goat anti-mouse antibody (1:500, ZSBG-bio, China) for 1 hour at room temperature. Cells treated with secondary antibody only were used as controls. The cell nuclei were stained with 5 μg/mL DAPI (Invitrogen) for 30 min at 37 ℃ and observation was conducted using confocal laser scanning microscopy (LSM700, Carl Zeiss). For flow cytometry, cells without nuclear staining were harvested using trypsin digestion and analyzed using an Attune® acoustic focusing cytometer (Life Technologies, CA) with 488 nm excitation. All of the experiments were carried out in triplicate.

Western blot assay

The quantitative analysis of integrin β_1 expression was conducted using a western blot assay (n = 3). The whole cell proteins were extracted from HCECs and RCECs using a RIPA lysis buffer (Beyotime, China) with protease inhibitors (Roche). The protein concentration was determined using a BCA protein assay kit (Lot#23225, Thermo Fisher). A total of 50 μg protein was used for SDS-PAGE with 10 % gel and was transferred to a nitrocellulose filter membrane (PALL) for blotting. The membrane was blocked with 5 % defatted milk for 1 hour at room temperature, incubated with primary mouse monoclonal anti-integrin β_1 antibody (1:200, ab30394, Abcam, USA) overnight at 4 ℃, and incubated with secondary horseradish peroxidase-conjugated antibody (1:5000, ZSBG-bio, China) for 1 hour at room temperature. The results were recorded and analyzed using a chemiluminescence imaging system (ChemiDoc MP, Biorad). All samples were assayed in triplicate.

***In vitro* nanomicelle binding study**

Human corneal epithelial cells (HCECs) were planted in glass microscopy dishes and incubated in complete medium. To facilitate observation, coumarin 6 was loaded into the micelles instead of FBP. After the cells grew to 70 % confluence, the cells were washed with PBS twice and pre-incubated with different endocytosis inhibitors to reduce the cell internalization of coumarin 6. Four endocytosis inhibitors were added into serum-free medium to pre-incubate the cells for 30 min at 37 ℃: 5 μM chlorpromazine (clathrin-mediated endocytosis inhibited), 8 μM dynasore hydrate

(dynamin-dependent endocytosis inhibited), 10 μM cytochalasin D (macropinocytosis-mediated endocytosis inhibited), and 100 μM genistein (caveolae-mediated endocytosis inhibited). Then, different formulations of nanomicelles were incubated with cells in complete medium with 1 μg/mL coumarin 6 for 2 min. The nanomicelle solution was removed and the cells were fixed. The cell membranes were stained with 5 μM Dil for 30 min. For the cRGD peptide competition study, cells were pre-incubated with excess cRGD peptide in 20 μg/mL for 30 min at 37 □ before treatment with endocytosis inhibitors. The experiment was assayed in triplicate and the nanomicelle binding properties were observed using confocal microscopy.

Trans-corneal penetration study using 3D-cultured multilayer HCEC spheroids

Multilayer corneal epithelium spheroids were constructed with HCECs using methods described in the literature.[3] Briefly, a 0.8 % agarose solution was freshly prepared and heated. The 96-well plates were coated with 50 μL of the agarose solution per well and sterilized for 30 min using UV irradiation. 100 μL of cell suspension (500 cells) were planted into each agarose-coated well and cultured in complete medium. The medium was changed every 2 days until the corneal epithelial spheroids grew to 200-250 μm diameter. To evaluate the trans-corneal penetration ability of different formulations, HCEC spheroids were incubated with free coumarin 6 (free C6), coumarin 6 nanomicelles (M-C6) and functional coumarin 6 nanomicelles (CTFM-C6) at a final concentration of 2 μg/mL coumarin 6 for 4 h (n = 5 spheroids). HCEC spheroids cultured with complete medium were used as the control. After treatment, the spheroids were rinsed with PBS and fixed with 4 % (wt/vol) formaldehyde solution for 20 min at room temperature. Then, spheroids were transferred into chambered coverslips and observed using two-photon confocal microscopy. Z-stack images were obtained by scanning the spheroids from top to bottom with 10 μm thickness and a total 60 μm depth (~15 images in each spheroid). To measure the penetration distance of each formulation, the spheroid image with the biggest diameter was selected and the penetration distance from spheroid edge to interior was measured using Adobe Photoshop CS 6 software. The relative penetration distance was defined as the ratio of nanomicelle penetration distances to free C6 penetration distances.

Animal experiments

All animal experiments were in compliance with guidelines for the care and use of laboratory animals and were approved by the institutional animal care and use committee of Wenzhou Medical

University and the National Center for Nanoscience and Technology of China. The New Zealand male albino rabbits and Sprague Dawley® rats (Charles River, China) were housed under standard conditions with free access to food and water.

Ocular surface retention study

The ocular surface retention study was conducted to evaluate the retention of nanomicelles after a single instillation. Sprague Dawley® rats (500-800 g) were randomly divided into three groups (n=5). In each group, 1.5 μL of either free C6, M-C6 or CFTM-C6 with a final C6 concentration of 2 μg/mL was instilled into the conjunctival sac of the left eye. The contralateral eye was used as untreated control. We used fluorescence microscopy for eye imaging at the initial instillation (0 h), 0.5 h, 1h, 2h, 3h and 4 h after instillation. The mean fluorescence intensities of areas inside the eyelid in each image were quantified by Image J software.

***In vivo* anti-inflammatory study**

To study the anti-inflammatory effect of the FBP formulations, ocular inflammation was induced by topical administration of sodium arachidonate (SA).[4-6] New Zealand albino male rabbits (2-2.5 Kg) were randomly divided into four groups (n = 6). In each group, 50 μL of 1 % SA solution (wt/vol) in PBS was instilled into the conjunctival sac of the left eye followed by closing of the eye for a few seconds. The contralateral eye was used as an untreated control. Either 50 μL PBS solution (the inflammatory positive control) or a nanomicelle formulation, including M-FBP and CTFM-FBP, were administered to the inflamed eye with an FBP concentration of 0.3 mg/mL twice, one dose instilled 30 min before ocular inflammation induction and the other instilled 30 min after ocular inflammation induction. Using the commercial Ocufen® eye drop (flurbiprofen sodium, 0.03 % wt/vol) as a reference, the FBNa formulation (flurbiprofen sodium, 0.03 % wt/vol) was prepared according to the formulation of commercial eye drops and administered to the inflamed rabbit eye in the same manner as the FBP nanomicelles. Examination of eyes was performed by a professional ophthalmologist blinded to the treatment using a slit lamp microscope 30, 60, 120 and 180 min after instillation of SA. The clinical ocular inflammation scores were recorded by evaluating the following parameters: iris hyperemia (grade from 0 to 2), conjunctiva congestion (grade from 0 to 3), swelling (grade from 0 to 4) and discharge (grade from 0 to 3). The corneal epithelial defects were examined using fluorescein staining 180 min after induction of ocular inflammation.

As an important ocular inflammatory feature, the polymorphonuclear (PMN) leukocytes in tears and aqueous humor were counted using a hemocytometer. For tear collection, the ocular surface of the rabbit eye was washed with 50 μL PBS from the upper eyelid to the conjunctiva three times and the tears and PBS were collected immediately. For aqueous humor collection, the rabbits were anesthetized with 2 % xylazine hydrochloride (wt/vol, 0.2 mL/Kg) and 3 % pentobarbital sodium (wt/vol, 1 mL/Kg) followed by topical anesthetic proparacaine hydrochloride eye drops (0.5 %, wt/vol). 150 μL of aqueous humor from each rabbit was withdrawn by anterior chamber paracentesis with an insulin syringe. The PMN leukocytes were quantified immediately after sample collection. All samples were assayed in triplicate and stored at -80 □ for ELISA measurement (n = 6).

ELISA

The changes in prostaglandin E_2 (PGE_2) and inflammatory cytokine IL-6 were measured using enzyme-linked immunoassay (ELISA). The mouse PGE_2 ELISA kit (R&D Systems, USA) and rabbit IL-6 ELISA kit (BD Biosciences, USA) were operated under manufacturers' instructions.

Intraocular pharmacokinetic study

Male New Zealand albino rabbits (2-2.5 Kg) were randomly divided into 3 groups (n = 4). A single dosage of 50 μL of an FBP formulation of FBNa, M-FBP and CTFM-FBP was instilled into the lower conjunctival sac of the rabbit eye at an FBP concentration at 0.3 mg/mL. At 0.5, 1, 2, 4, 6, 9, 12 and 24 h, the rabbits were anaesthetized and 20 μL of aqueous humor was withdrawn by anterior chamber paracentesis. The FBP concentration in the aqueous humor samples was quantified using the HPLC method described above. To prepare the samples for HPLC analysis, 20 μL aqueous humor was mixed with 80 μL acetonitrile and proteins were removed by centrifugation.[7, 8] The relative bioavailability (Fr) of M-FBP and CTFM-FBP versus the FBNa formulation was calculated using the following formula:

$$Fr = AUC_1/AUC_0 \times 100\ \%$$

where AUC_1 is the area under curve (AUC) of M-FBP or CTFM-FBP and AUC_0 is the AUC of the FBNa formulation.

Ocular tolerance study

The potential ocular irritancy and toxicity of the FBP nanomicelle formulation were evaluated using a modified Draize test.[9] Briefly, the New Zealand male albino rabbits were randomly divided into five groups (n = 3). 50 μL of PBS (irritant negative control), 1 % SDS (wt/vol, irritant positive

control), and three FBP formulations (FBP concentration of 0.3 mg/mL) were instilled into the left eye of rabbits every 30 min for 6 h. At 10 min, 6 h and 24 h after the last instillation, all eyes were examined using a slit lamp microscope and scored according to the scoring system above. In the last examination at 24 h, all experimental eyes were evaluated using optical coherence tomography (OCT) and H&E staining for histological examination. All experiments were carried out in triplicate.

OCT examination

A custom-built ultra-high resolution OCT (UHR-OCT) instrument provided by Wenzhou Medical University was used to assess changes of topographic thickness of the cornea and retina. Male New Zealand albino rabbits were anaesthetized prior to OCT examination. For the corneal imaging, the rabbits were fixed in front of a slit lamp on which the UHR-OCT probe was equipped and the eyes were kept open with the lids gently held apart. Images were captured after the operator adjusted the vertex at the center of the OCT image to maximize the vertex reflection. For the retina imaging, a 90 diopter (D) ocular lens (Volk Optical, USA) was placed onto the slit lamp. The retina near the optic nerve head was identified through infrared light illumination and images were captured with a scan width of 8.021 mm for the horizontal meridian and 7.989 mm for the vertical meridian. The cornea and retina UHR-OCT images were analyzed using a custom software J-OCT and the central corneal and retinal thicknesses were quantified.

Histological examination

To evaluate the influence of different formulations on normal eye tissues, the New Zealand albino rabbits were sacrificed by intravenous air injection after the ocular tolerance experiment and the eyes were enucleated immediately. The eye balls were cleaned and washed with PBS and fixed in 10 % formalin solution (vol/vol) for 2 days. Then, the eye balls were dehydrated in a gradient alcohol series and embedded in paraffin. 5 μm cross sections were cut and stained with hematoxylin and eosin (H&E) for histopathological evaluation (~5 slices for each eye).

Statistical analysis

All data are presented as mean ± s.e.m. in this work. Statistical analysis was conducted using the Graphpad Prism software. Statistical significance was determined by Student's *t*-test, one-way ANOVA and two-way ANOVA test set at $P < 0.05$.

Data availability

All data in this manuscript are available from corresponding authors upon request.

Author contributions

Y.H.W., X.W.M. and X.J.L designed the study. Y.H.W. performed characterization of nanomicelles, animal experiments, and wrote the manuscript. X.W.M performed major cell biology experiments, contributed to the discussion, and revised the manuscript. J.C. performed preparation of nanomicelles and contributed to the discussion. Y.Q.W. and K.H.N contributed to the ophthalmic experimental techniques and part of animal tests. H.C. contributed to communication and coordination work. C.L, J.L, S.Z.C, Y.L.G. and Z.B.H contributed to the data analysis. X.J.L. contributed to designation, discussion and supervision of the project. All authors reviewed and commented on the manuscript.

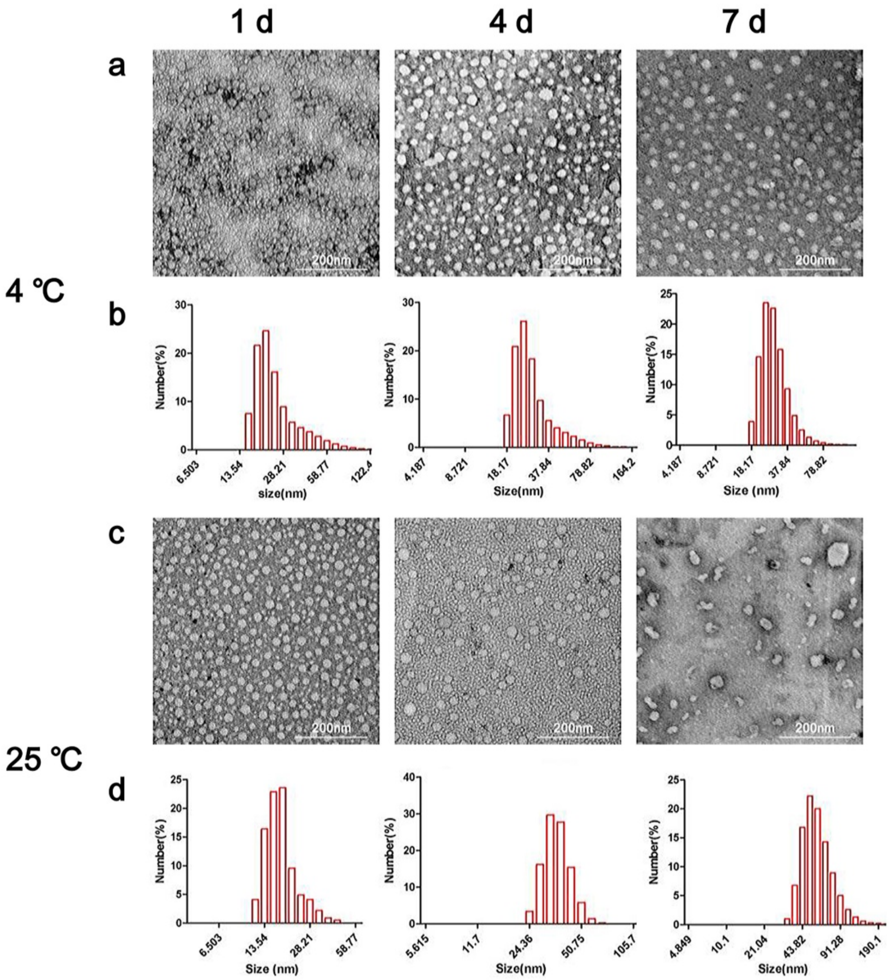

Figure S1. Stability of CTFM-FBP nanomicelles in artificial tears. a) TEM images and b) DLS analysis of CTFM-FBP stored in artificial tears from 1 day to 7days under 4 □. c) TEM images and d) DLS analysis of CTFM-FBP stored in artificial tears from 1 day to 7days under 25 □.

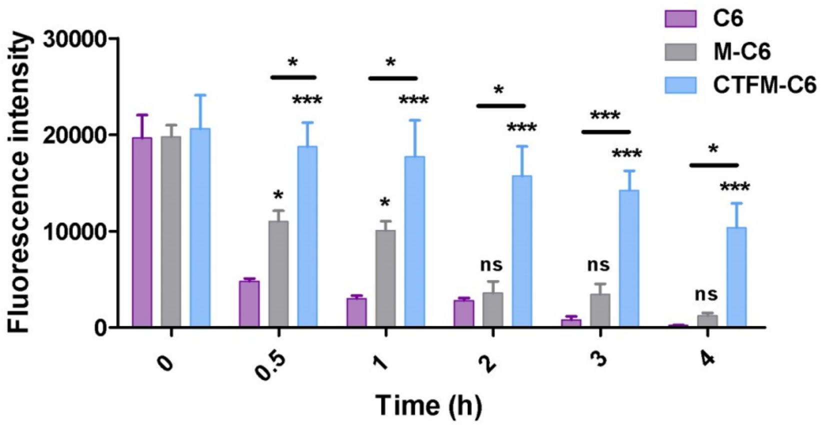

Figure S2. Quantitative analysis of fluorescence intensity on ocular surface after eyes were treated with different C6 formulations. The free C6, M-C6 and CTFM-C6 formulation was instilled into rats' eyes for only once. At different time intervals, the fluorescence images of eyes were taken by fluorescence microscopy. Quantitative analysis of fluorescence intensity was measured using Image J software. The data are shown as the mean ± s.e.m. Statistical analysis of one-way ANOVA was conducted by comparing with the free C6 group (n = 5). *P<0.05, **P<0.01, ***P<0.001.

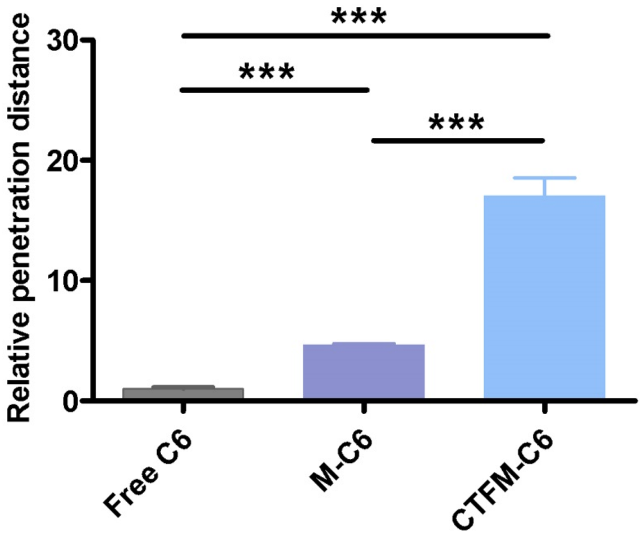

Figure S3. Relative penetration distance of different C6 formulations in HCEC spheroids. HCEC spheroids were co-cultured with free C6, M-C6 and CTFM-C6 for 4 hours. Visualization of the spheroids was conducted using confocal microscopy. To measure the relative penetration distance, the spheroid image with the biggest diameter was selected and the penetration distance from spheroid surface to interior was measured using Adobe Photoshop CS6 software. The quantitative analysis showed that CTFM-C6 exhibited significantly more penetration distance into the HCEC spheroid than M-C6 and free C6. The data are shown as the mean ± s.e.m. ***P<0.001, unpaired Student's *t*-test (n = 5).

Table S1 Characterization and physicochemical properties of FBP nanomicelle formulations. The data are shown as the mean ± s.e.m. (n = 3).

**Formulation**	**Size (nm)**	**PDI**	**Zeta potential (mV)**	**EE (%)**	**DLC (%)**
M-FBP	19.1 ± 1.8	0.20 ± 0.07	-25.2 ± 0.98	89.94 ± 1.36	8.05 ± 0.02
CTFM-FBP	19.3 ± 2.1	0.20 ± 0.04	-21.97 ± 0.67	90.0 ± 0.02	8.42 ± 0.22

Table S2 Clinical scores of ocular inflammation in the iris of rabbit eyes, examined using a slit lamp (n = 6).

**Time (h)**	**Formulation**																							
	**PBS**						**FBNa**						**M-FBP**						**CTFM-FBP**					
0.5	1	1	1	1	2	1	1	1	1	1	1	1	1	1	1	1	0	1	0	0	0	1	0	1
1	1	0	1	1	1	1	0	1	1	1	1	1	0	0	1	1	1	1	0	0	0	0	1	0
2	1	0	2	1	1	1	0	1	1	1	1	1	0	0	0	1	1	1	0	0	0	0	0	1
3	1	0	1	1	1	1	0	0	0	1	1	1	0	0	0	0	1	1	0	0	0	0	0	0

Table S3 Pharmacokinetic parameters of different FBP formulations in aqueous humor after ophthalmic instillation (n = 4). Statistical analysis was conducted using Student's t-test.

**Formulation**	**Cmax (μg/mL)**	**AUC (μg/mL·h)**	**Fr (%)**
FBNa	0.52 ± 0.12	1.10 ± 0.28	__
M-FBP	1.02 ± 0.25*	6.27 ± 1.24***	570
CTFM-FBP	2.02 ± 0.62**$^{\#}$	11.50 ± 1.95***$^{\#\#}$	1045.45

* Statistically difference to the FBNa group, *P<0.05, **P<0.01, ***P<0.001.

$^{\#}$ Statistically difference to the M-FBP group, $^{\#}$ P<0.05, $^{\#\#}$P<0.01.

Table S4 Ocular irritant scores of different formulations evaluated using a modified Draize test (n = 3). Five groups of New Zealand albino rabbits were instilled with PBS, 1% SDS, FBNa, M-FBP or CTFM-FBP 12 times. Ocular irritant scores were recorded 10 min, 6 h and 24 h after the last instillation.

**Formulations**	**10min**		**6h**		**24h**	
	**Conjunctiva**	**Iris**	**Conjunctiva**	**Iris**	**Conjunctiva**	**Iris**
PBS	0	0	0	0	0	0
	0	0	0	0	0	0
	0	0	0	1	0	0
SDS	8	1	3	1	0	0
	8	0	2	0	0	0
	6	0	3	0	0	0
FBNa	1	0	0	0	0	0
	1	0	0	0	0	0
	0	0	0	0	0	0
M-FBP	1	0	0	0	0	0
	0	0	0	0	0	0
	0	0	0	0	0	0
CTFM-FBP	0	0	0	0	0	0
	0	0	0	0	0	0
	1	0	0	0	0	0

References

[1] E.P. Shen, R.-Y. Tsay, J.-S. Chia, S. Wu, J.-W. Lee, F.-R. Hu, *Invest Ophth Vis Sci*, **2012,** *53*, 6416.

[2] K.Y. Chan, R.H. Haschke, Isolation and culture of corneal cells and their interactions with dissociated trigeminal neurons, *Exp Eye Res*, **1982,** *35,* 137.

[3] K. Huang, H. Ma, J. Liu, S. Huo, A. Kumar, T. Wei, X. Zhang, S. Jin, Y. Gan, P.C. Wang, *ACS nano*, **2012**, *6*, 4483.

[4] A. Vasconcelos, E. Vega, Y. Perez, M.J. Gomara, M.L. Garcia, I. Haro, *Int J Nanomed*, **2015**, *10*, 609.

[5] A.K. Sharma, P.K. Sahoo, D.K. Majumdar, A.K. Panda, *Nanotechnol Rev*, **2016**, *5*, 435.

[6] L.D. Waterbury, A.J. Flach, *J Ocul Pharmacol Th*, **2004**, *20*, 345.

[7] S. Shi, Z. Zhang, Z. Luo, J. Yu, R. Liang, X. Li, H. Chen, *Sci Rep*, **2015**, *5*, 11337.

[8] Z. Zhang, Z. He, R. Liang, Y. Ma, W. Huang, R. Jiang, S. Shi, H. Chen, X. Li, *Biomacromolecules*, **2016**, *17*, 798.

[9] R. Pignatello, C. Bucolo, G. Spedalieri, A. Maltese, G. Puglisi, *Biomaterials*, **2002**, 23, 3247.
